# Dietary Risk Factors Associated with Development of Gastric Cancer in Nepal: A Hospital-Based Case-Control Study

**DOI:** 10.1155/2020/5202946

**Published:** 2020-06-03

**Authors:** Sunil Kumar Shah, Dev Ram Sunuwar, Narendra Kumar Chaudhary, Pushpa Rai, Pranil Man Singh Pradhan, Narayan Subedi, Madhu Dixit Devkota

**Affiliations:** ^1^Public Health and Nutrition, Bagmati Welfare Society Nepal, Sarlahi, Nepal; ^2^Department of Nutrition and Dietetics, Nepal APF Hospital, Kathmandu, Nepal; ^3^Department of Radiology, Nepal Orthopaedic Hospital, Kathmandu, Nepal; ^4^Department of Emergency, Patan Academy of Health Sciences, Lalitpur, Nepal; ^5^Department of Community Medicine, Institute of Medicine, Tribhuwan University, Kathmandu, Nepal; ^6^Central Department of Public Health, Institute of Medicine, Tribhuwan University, Kathmandu, Nepal; ^7^Upendra Devkota Memorial National Institute of Neurological and Allied Sciences, Kathmandu, Nepal

## Abstract

**Objectives:**

Gastric cancer (GC) is one of the most prevalent neoplasms and a leading cause of mortality globally. To our knowledge, its relationship with dietary factors is not adequately studied and understood in the Nepalese context. This study is aimed at exploring the relationship between the possible dietary risk factors responsible for gastric cancer in Nepal.

**Methods:**

A hospital-based matched case-control study was conducted in two specialized cancer hospitals in Nepal. A total of 237 participants (79 cases and 158 controls) were included in the study. Patients diagnosed within one year (incidence case) with histologically confirmed gastric cancer were taken as cases, and healthy visitors of gastric and nongastric cancer patients without past and present history or not a suspected information of gastric cancer were included as controls. A face-to-face interview was conducted using a semi-structured food frequency questionnaire. Backward stepwise conditional logistic regression was used to estimate the magnitude of the association between the independent variables and gastric cancer. Results were presented as the crude odds ratio (COR) and adjusted odds ratio (AOR) with 95% confidence intervals (CI). A *P* value < 0.05 was considered statistically significant.

**Results:**

In the adjusted multivariable conditional logistic regression model, an increased risk of gastric cancer was more likely to have higher odds among those respondents who had a high consumption of processed meat (AOR = 3.99, 95% CI: 0.90-17.66), preferences of a high amount of fats/oil (AOR = 4.64, 95% CI: 1.56-13.72), and preferences of high amounts of salts (AOR = 4.18, 95% CI: 1.30-13.44). Conversely, those respondents who consumed higher amounts of fruits (AOR = 0.21, 95% CI: 0.07-0.65) were seen to have lower odds of gastric cancer.

**Conclusions:**

Our study found an increased risk of gastric cancer with frequent consumption of red meat, processed meat, high preferences of salt, fats/oil, and condiments. Regular consumption of fruits had a protective effect against gastric cancer. Providing nutrition education, public awareness, and lifestyle modification are primary steps to promote the avoidance of risk factors and change unhealthy dietary habits to prevent gastric cancer in Nepal.

## 1. Introduction

Cancer is the most leading cause of morbidity and mortality globally [1]. Out of all cancers, gastric cancer is one of the most common and fatal cancer considering intractable public health challenge in the world [2]. According to the Global Cancer Incidence, Mortality, and Prevalence (GLOBOCAN) 2018 report, gastric cancer is the 5^th^ most common cancer and the 3^rd^ most leading deadly cancer across the globe. About 783,000 (8.2% of all cancer) deaths were reported in 2018 globally [1]. American Cancer Society estimates the 606,520 deaths from cancer, among them 27,600 will be new stomach cancer cases and there will be 11,010 deaths only in the United States in 2020 [3]. Likewise, more than 70% of the total gastric cancer occurs in developing countries and more than 50% of cases occur in eastern Asia. It is the third most common cancer after breast and lung cancer. Similarly, it is the second most common cause of cancer death after lung cancer in Asia [4]. Also, gastric cancer is the second leading cause of cancer mortality in India [5]. There is no exact prevalence of cancer in Nepal because of the unavailability of population-based national cancer study [6]. However, Kandel et al. have reported that gastric cancer is the fourth most common cause of cancer related deaths in Nepal [7]. A study based on cancer registries in different hospitals has indicated that gastric cancer is the second most common cancer after lung cancer related to death in the male in Nepal [8].

Development of gastric cancer is a complex, multifaceted, and diverse risk factors [9]. The epidemiological study suggested that many modifiable and non-modifiable risk factors such as environmental factors, lifestyle factors, and infection by *Helicobacter pylori* (*H. pylori*) contribute to cause gastric cancer [10]. Among them, many modifiable risk factors play an important role in the development of gastric cancer in many people. The risk factors consist of cigarette smoking, heavy alcohol consumption, overweight/obesity, high sodium intake, high red meat consumption, and low fruits and vegetable intake [11, 12]. Western dietary patterns have long been considered an important risk factor for gastric cancer [13]. Red and processed meat consumption has significantly increased globally in recent years resulting to the likelihood of risk of gastric cancer [14, 15]. Processed meat includes foods preserved by salting, smoking, or adding nitrates or nitrite [16]. Diets high in salt could damage the gastric mucosa, leading to gastritis, increased DNA synthesis, and excessive cell replication [17, 18]. Processed meat often contains, besides high amounts of salts, carcinogenic N-nitroso compounds [17]. Nevertheless, the precise contributions of red and processed meat on gastric cancer are still in dispute because of insufficient data [15]. On the other hand, the Mediterranean diet has been found to protect from gastric cancer [19]. The Mediterranean diet is characterized by frequent consumption of fruits, vegetables, complex carbohydrates, pulses, fish, and low consumption of meat and cheese [20].

The prevalence of stomach cancer among males and females were 9.3% and 5.7%, respectively, in Nepal [21]. Cancer treatment, prevention, and control are slowly improving in Nepal. Steady progress has been achieved to some extent in the past two decades, despite many socioeconomic and political conditions [22]. Indeed, cancer treatment is very costly in Nepal requiring expensive facilities, highly specialized health personnel and expensive drugs [23]. Patients from under the poverty line can hardly afford this kind of expensive treatment. The government of Nepal has tried to give subsidy in the cancer treatment. However, there is no existing government policy for the prevention of cancer. To the best of our knowledge, the association of dietary factors with the risk of gastric cancer has not yet been examined in the context of Nepal. Therefore, this study is aimed at providing empirical evidence for the association of different dietary factors with the risk of gastric cancer in the Nepalese context.

## 2. Materials and Methods

### 2.1. Study Design

The study was a hospital-based matched case-control study conducted in two specialized cancer hospitals: Bhaktapur Cancer Hospital (BCH) and B.P. Koirala Memorial Cancer Hospital (BPKMCH) in Nepal. These hospitals cover two thirds (49% and 18% from BPKMCH and BCH, respectively) of total gastric cases reported in Nepal [24]. BPKMCH is the first national cancer hospital with high patient flow referred from most of the districts in Nepal [25]. The study was conducted from June 2016 to March 2017.

### 2.2. Cases

Cases were the patients who were diagnosed as gastric cancer with histologically or cytological confirmed within one year (incidence case) preceding the interview. They were included in the study while they were attending in out- and inpatient departments of these two hospitals. The patients who had been already diagnosed with cases of gastric cancer for more than one year preceding interview, patients who were not able to respond due to any kind of unfavorable circumstances, patients having any chronic systemic diseases particularly affecting dietary patterns, and pregnant and lactating women were excluded from the study. The cases for which matched control age and sex not found were excluded from the study.

### 2.3. Controls

Subjects in the control groups were selected from healthy visitors (patients' relatives) of gastric and nongastric cancer patients attending to the same hospital with no past and present history or not a suspected information of gastric cancer. For the selection of control groups, we did not conduct any diagnostic confirmatory tests for control groups and information was collected verbally from the respondents based on past and present history of not having gastric cancer. The matching was done with cases by age (+5 years interval) and sex. The ratio of the size of the case and control group was taken 1 : 2. The same exclusion criteria for cases were applied to the control groups. In addition, the person who did not have any malignancy history and those who are not following special diets, such as those who underwent a weight reduction diet plan, were included in the control groups.

### 2.4. Sampling Strategy and Setting

Two specialized cancer hospitals were selected purposively. As this study was hospital-based, we used the consecutive sampling method until and unless the sample size was met. All confirmed gastric cancer cases found in out- and inpatient department of the hospital were obtained as a study sample. The sample size was calculated based on a study done by Ward and Lopez-Carillo [26]. After adjusting a 10% nonresponse rate, the final sample size was 237 (79 participants in cases and 158 participants in the control group, respectively) at 95% confidence interval and 90% power. The sample size was calculated using Epi Info version 7 (StatCalc). The response rate for the case and control groups was 100% and 96%, respectively.

The trained enumerator collected data from all participants who gave informed consent to participate in the study. All cases and controls were interviewed approximately 20-30 minutes during the visit to out- and inpatient departments of the hospital. Data on cases and controls were collected at the same time and interviewed in the same settings. A face-to-face interview was performed to collect data using the semi-structured questionnaire. Data were collected regarding sociodemographic characteristics of the respondents, lifestyle factors, clinical history, and dietary habits. For the validity of sociodemographic variables, the questionnaire was adopted from Nepal Demographic and Health Survey (NDHS) 2011 [27] and STEP Survey 2013 [28]. A semiquantitative food frequency questionnaire (SQFFQ) was used to assess the dietary habits. The FFQ was developed specifically from the previous similar study done in a different time in different countries [29, 30]. Similarly for the validity of dietary factors, food groups were adopted from FANTA [31] and the categorization of food was based on STEP Survey 2013 [28]. A detailed dietary history was obtained from each participant, consumed individual food items (frequency of consumption) in one year preceding their gastric cancer (cases) diagnosis or preceding year (control). Also, the present dietary habit was asked to rule out possible changes in diet, based on this consideration, this reflects the lifetime dietary habit of the study participants. The frequency of consumption was measured on a 5-grade scale: (1) never, (2) less frequently (<3 times/month), (3) 1-2 times/week, (4) ≥3 times/week, and (5) once daily [29]. Intake of individual foods was categorized into dichotomous variables (high and moderate/low) for further analysis. We dichotomized the variables: high, daily or 1-2 times/week or ≥3 times/week, and moderate/low, never or less frequently (<3 times/month), respectively. We included lifestyle factors such as the history of tobacco, smoking, and alcohol consumption. Participants who had consumed alcohol regularly or 1-3 times/week were regarded as ever drinker otherwise never a drinker. Likewise, participants who had smoked cigarettes, cigars, or pipes regularly for 1-3 times/week at the time of interview were considered a smoker otherwise never a smoker. The questionnaire was translated into Nepali language, and pretesting was done in 5% of the study sample, i.e., 12 (4 cases and 8 controls) in Bhaktapur Cancer Hospital to ensure the reliability of the questionnaire.

Ethical approval was obtained from the Institutional Review Board (IRB) of the Institute of Medicine (IOM) (reference number 117 (6-11-E)/2073/2074). The aim of the study was informed to the participants. If they agreed, written informed consent was taken from those participants before the interview. Confidentiality and privacy of the participants were maintained by not sharing the individual information.

### 2.5. Statistical Analysis

Data were entered in EpiData version 3.2 and transferred to STATA/MP 14.1 (StataCorp LP, College Station, Texas) for statistical analysis. Predictor variables were recoded and dichotomized to performed analysis. A descriptive analysis was performed for each variable. Frequency and percentage were computed for a categorical variable, whereas mean and standard deviation was computed for continuous variables. All initial models were run with all potential factors associated with gastric cancer in the bivariate analysis. Before conducting multivariable analysis, multicollinearity was tested between the predictors' variables to examine and reported any collinearity using variation inflation factor (VIF) with a cutoff point < 10 and tolerance test with a cutoff < 1 [32]. In multivariable analysis, backward stepwise methods were used which led to the removal of insignificant variables (*P* > 0.05), resulting in a parsimonious model. Conditional logistic regression was used to identify the relationship between dietary factors and gastric cancer with adjusting confounding variables. We used the Wald test statistics for the significance test of the covariate (STC) for the selection of confounder [33]. Results were presented as the crude odds ratio (COR) and adjusted odds ratio (AOR) with 95% confidence intervals (CI). A *P* value < 0.05 was considered statistically significant.

## 3. Results and Discussion

### 3.1. Results

In the current study, we included the 237 participants (79 cases and 158 control) in the ratio of 1 : 2.  Table 1 depicts the sociodemographic characteristics of respondents for both case and control groups. The mean (± SD) age of respondents in both case and control groups was 56 (± 12) and 53 (± 11) years, respectively. This study found that more than half of respondents (70% cases and 60% controls) are ≥50 years of age. About more than half of the participants (62% cases and 58% controls) were male in both case and control groups, respectively. The majority of the respondents (80%) attended at least primary and lower levels of education in case groups whereas more than half of the participants (60%) had a primary and lower level of education in control groups. Similarly, about 70% of the cases and 44% of the control group were from a rural region. Most of the participants were from the Hindu religion in both groups (90% cases and 80% controls). Approximately two-thirds of the participants (67%) had involvement in agricultural occupation in the case groups whereas slightly more than half of the participants (54%) had involvement in the agriculture sector in control groups (Table 1).

Table 2 shows the bivariate and multivariable conditional logistic regression models for dietary factors and the risk of gastric cancer. In the bivariate conditional logistic regression model, several factors were more likely to have higher odds of suffering from gastric cancer: high consumption of process meat compared to moderate/low consumption (COR = 10.27, 95% CI: 4.33-24.37), high preferences of fats/oils compared to moderate/low preferred (COR = 8.90, 95% CI: 4.16-19.05), high intake of salts compared to moderate/low preferences of salts (COR = 8.01, 95% CI: 3.70-17.32), high preferences of condiments compared to moderate/low preferences of condiments (COR = 6.60, 95% CI: 3.01-14.48), consumption of high amounts of red meat compared to moderate/low amounts of red meat (COR = 4.80, 95% CI: 2.41-9.58), consumption of alcohol compared to no consumption of alcohol (COR = 2.29, 95% CI: 1.31-4.00), consumption of high amounts of white meat compared to moderate/low preferences of white meat (COR = 2.05, 95% CI: 1.13-3.73), habit of tobacco used compared to no tobacco used (COR = 1.94, 95% CI: 1.07-3.53), and consumption of high amounts of green compared to moderate/low amounts of green vegetablesk (COR = 1.82, 95% CI: 1.04-3.19. On the other hand, respondents who had a consumption of high amounts of fruits compared to moderate/low amounts of fruits(COR = 0.11, 95% CI: 0.05-0.24) and refrigerator usedcompared to no refrigerator used (COR = 0.30, 95% CI: 0.14-0.64) were found likely to lower odds of suffering from gastric cancer.

After adjusting the confounding factors in the multivariable conditional logistic regression model (Table 2), respondents who had a high consumption of processed meat compared to moderate/low consumption (AOR = 3.99, 95% CI: 0.90-17.66), preferences of a high amount of fats/oil compared to moderate/low preferences (AOR = 4.64, 95% CI: 1.56-13.72), and preferences of high amounts of salts compared to moderate/low preferences of salts (AOR = 4.18, 95% CI: 1.30-13.44) were more likely to risk of gastric cancer. However, respondents who consumed higher amounts of fruits compared to moderate/low amounts of fruits (AOR = 0.21, 95% CI: 0.07-0.65) were seen to have defended against the risk of gastric cancer.

### 3.2. Discussion

The present study found that patients who had a consumption of processed meat, preference of high amount of fats/oils, and consumption of the high amount of salt were more likely to suffer from gastric cancer. In the adjusted model, the current study depicted the consumption of white meat as a protective measure for the prevention of gastric cancer. In contrast, red meat and processed meat contributed in causing gastric cancer. This finding is consistent with the meta-analysis done by Kim et al. which indicates that the consumption of white meat decreases the risk of gastric cancer by 20% while the red and processed meat increases the risk of gastric cancer by 41% and 57%, respectively [15]. Likewise, this result is consistent with the study done by Cross et al. which found that the red and process meats are likely to increase the risk of gastric cancer. The plausible biological mechanism for this is that the involving iron, heterocyclic amines, polycyclic aromatic hydrocarbons, and N-nitroso compounds and haem iron are abundantly present in meat which promotes the endogenous formation of carcinogenic N-nitroso compounds (NOCs) [17, 34]. NOCs lead to the DNA damage which supports the friendly environment for the growth of *H. pylori* [35], and *H. pylori* are the serious leading factor for facilitating gastric cancer [36]. However, a prospective cohort study done in the Netherlands found that red and processed meat consumption is positively associated with an increased risk of esophagus cancer but not with gastric cancer [18]. Furthermore, a meta-analysis done by Susanna et al. depicted that the increased consumption of processed meat is associated with gastric cancer [16]. Likewise, in processed meat, the processing, storage, and cooking methods are likely to increase the risk of gastric cancer. This could be due to the heterocyclic amines and polycyclic aromatics hydrocarbons produced when meat is cooked at a high temperature [15]. On the other hand, the consumption of more white meat was positively associated with lowering the risk of gastric cancer. The plausible mechanism could be the lesser haem iron in the white meat which resists an increased risk of gastric cancer because it contributes to the suppression of endogenous formation of N-nitroso compounds (NOCs) [37]. Similarly, white meat is the good source of polyunsatuared fatty acids (PUFAs) which contains a lower level of cholestrerol and haem iron [15].

The present study showed the positive association of gastric cancer with high consumption of salt. Systematic review and meta-analysis of case-control studies exhibited the positive association of dietary salt intake and gastric cancer [38]. The possibility of the biological mechanism was explained by Galvan-Portillo MV et al. [10] who found the induction of gastric atrophy on mice with the utilization of high intake of salt. In turn, it enhances colonization [10]. Furthermore, high dietary salt which was contained in cured or salted meat products injured gastric mucosa and induced significant gastric pathological mechanism and inflammation [39]. Likewise, *H. pylori* is the major risk factor for causing gastric cancer. So, increasing consumption of salt may likely to flourish the *H. pylori*- associated carcinogenesis through proliferation, pit cell hyperplasia, and granular atrophy [36].

The preference for high consumption of fat-/oil-containing food caused higher odds of gastric cancer than that of low/moderate consumption of fat. The study of Nishimoto et al. also favors this finding [30]. A meta-analysis of observational studies depicted that the consumption of fat is positively associated with the risk of gastric cancer [9]. Similarly, the present study found that the consumption of high amounts of condiments is likely to cause gastric cancer in the unadjusted model. This result is consistent with the study conducted by Galván-Portillo et al. that showed the significant association between chilly consumption and gastric cancer [10].

In the present study, the frequent consumption of fruits has decreased the chance of having gastric cancer. The risk of gastric cancer was lowered by 40% among those who consumed fruits daily as compared to those who consumed less than one day in a week, and similarly, the risk of gastric cancer was lowered by 30% among those who consumed green vegetables daily [30]. In line with the previous studies, these findings are consistent with Turati et al. and Wang et al. who reported the consumption of more fruits and vegetables was likely to reduce the risk of gastric cancer compared to that of low fruit consumer [20, 40]. The favorable effect of fruits and green vegetables against gastric cancer has been related to several factors. Indeed, fruits and vegetables are adequate sources of micronutrients and other bioactive components, including carotenoids; folate; vitamins C, D, and E; flavonoids; dietary fibers; and selenium. These factors may act against the anticancer role through their antioxidative activities, free-radical trapping capacity, modulation of detoxification enzymes, antimutagenic and antiproliferative properties, and stimulation of the immune system [20, 41].

The present study revealed that the refrigerator uses were found to be of caring against gastric cancer in the bivariate analysis. This finding is reliable with Munoz et al., who found that the risk of gastric cancer is decreased by 30% with the refrigerator use [42]. In the current study, tobacco use increased the threat of gastric cancer in the nonadjusted model. A multiethnic cohort study was done over 215,000 men and women, who represented from different five ethnic groups (African Americans, Japanese Americans, Latin Americans, Native Hawaiians, and Whites) also found the positive association of gastric cancer with tobacco use [43]. Moy et al. suggested that tobacco smoking are more likely to affect gastric cancer compared with nonsmoker [44]. Tobacco consists of many carcinogens like N-nitroso compounds. These compounds may bind to the gastric mucosa and ultimately increase the risk of dysplasia and intestinal metaplasia which are the precursor lesion of gastric cancer [43]. Our study prevailed positive association of gastric cancer with alcohol consumption in bivariate analysis but not in the adjusted model. Moy et al. remarked that about four drinks or more per day consumption increases the probability of gastric cancer [44]. Ethanol present in alcohol acts as a solvent for tobacco that enhances the penetration of tobacco gastric carcinogens and nitrosamines, and finally, the nitrosamines lead to gastric cancer [45]. Besides these, the ethanol is metabolized to acetaldehyde which is a group one carcinogen and that carcinogen is ultimately oxidized into nontoxic acetate. Hence, the occurrence of gastric cancer among heavy drinkers is 20% more than nondrinkers [45].

Our study had several limitations. Recall bias might have occurred in the selection of healthy visitors of gastric and nongastric cancer patients as control groups because they were selected only on the basis of present and past history given verbally. We did not collect the information about the well-established confounding factor *H. pylori* infection in this study. Despite these limitations, the consumption of antioxidant-rich diet such as vitamin C contributed to a protective effect from gastric cancer among *H. pylori*-infected patients [46]. Two different studies suggested that the H. pylori antibody was seen to be positive only among gastric noncardia cancer subjects who consumed processed meat; however, the association was statistically insignificant [18, 47]. Similarly, our study did not differentiate the type of gastric cancer (cardia and noncardia cancer) and histological subtype (intestinal) and diffuse (undifferentiated). The sample size of our study was small; therefore, findings cannot identify the rigorous causal association between dietary factors and GI cancer and cannot be generalized to a wider population. Despite these few limitations, our study had some strengths. This study was a well-defined matched case-control study where the cases and controls were selected among similar age categories.

## 4. Conclusions

The risk of gastric cancer is associated with the frequent consumption of red meat, processed meat, and high intake of salt, fats/oil, and condiments. Everyday consumption of fruits had a protective effect on the prevention of gastric cancer. Providing nutrition education, public awareness, and lifestyle modification could be the primary steps to promote the avoidance of risk factors and change unhealthy dietary habits to prevent gastric cancer in Nepal. Further studies with a larger sample size, representing diverse population including well-designed dietary assessment and strict control of confounders, are warranted to identify the association between dietary factors and the risk of gastric cancer.

## Figures and Tables

**Table 1:** Sociodemographic characteristics of the respondents.

Variables	Case (*n* = 79)		Control (*n* = 158)		*P* value^1^
	*n*	%	*N*	%	
Age (years) mean ± SD	56 ± 12		53 ± 11		
Age category					
<50 years	24	30	63	40	0.153
≥50 years	55	70	95	60	
Sex					
Male	49	62	92	58	0.575
Female	30	38	66	42	
Ethnicity					
Brahmin/Chhetri	26	33	60	38	0.746
Janajati/Newar	22	28	41	26	
Dalit/others	31	39	57	36	
Religion					
Hindu	71	90	130	83	0.149
Others	8	10	27	17	
Education					
Primary and lower	63	80	99	63	0.008∗
Secondary and above	16	20	59	37	
Occupation					
Agriculture	53	67	86	54	0.127
Business/service	13	16	43	27	
Labor/others	13	17	29	19	
Income					
NRs ≤ 15000/months	50	63	86	54	0.193
NRs > 15000/months	29	37	72	46	
Residence					
Rural	55	70	69	44	<0.001∗
Urban	24	30	89	56	

^1^ *P* value for chi-squared test. ^∗^Statistically significant at *P* < 0.05. 1USD = 115NRs.

**Table 2:** Association of dietary and lifestyle factors with the risk of gastric cancer.

Variables	Case (*n* = 79)	Control (*n* = 158)	Bivariate analysis	Multivariable analysis
	*n* (%)	*n* (%)	COR (95% CI)	AOR (95% CI)^¥^
Refrigerator				
Yes	11 (14)	53 (34)	0.30 (0.14-0.64)∗∗	0.56 (0.16-1.91)
No	68 (86)	105 (66)	Ref	Ref
Tobacco use				
Yes	50 (63)	77 (49)	1.94 (1.07-3.53)	
No	29 (37)	81 (51)	Ref	
Consumption of alcohol				
Yes	41 (52)	50 (32)	2.29 (1.31-4.00)∗∗	0.67 (0.23-1.93)
No	38 (48)	108 (68)	Ref	Ref
Consumption of white meat^a^				
High	53 (67)	81 (51)	2.05 (1.13-3.73)∗∗	0.43 (0.12-1.47)
Moderate/low	26 (33)	77 (49)	Ref	Ref
Consumption of red meat^a^				
High	40 (51)	32 (20)	4.80 (2.41-9.58)∗∗∗	3.16 (0.86-11.51)
Moderate/low	39 (49)	126 (80)	Ref	Ref
Consumption of processed meat^a^				
High	63 (80)	60 (38)	10.27 (4.33-24.37)∗∗∗	3.99 (0.90-17.66)∗
Moderate/low	16 (20)	98 (62)	Ref	Ref
Consumption of dairy product^a^				
High	66 (84)	126 (80)	1.30 (0.63-2.67)	
Moderate/low	13 (16)	32 (20)	Ref	
Preferences of amount of fats/oil^a^				
High	48 (61)	26 (16)	8.90 (4.16-19.05)∗∗∗	4.64 (1.56-13.72)∗∗
Moderate/low	31 (39)	132 (84)	Ref	Ref
Preferences of amount of salts^a^				
High	37 (47)	15 (10)	8.01 (3.70-17.32)∗∗∗	4.18 (1.30-13.44)∗∗
Moderate/low	42 (53)	143 (90)	Ref	Ref
Preferences of condiments^a^				
High	32 (41)	16 (10)	6.60 (3.01-14.48)∗∗∗	1.28 (0.38-4.34)
Moderate/low	47 (59)	142 (90)	Ref	Ref
Consumption of green vegetables^a^				
High	43 (54)	63 (40)	1.82 (1.04-3.19)∗	1.78 (0.66-4.82)
Moderate/low	36 (46)	95 (60)	Ref	Ref
Consumption of fruits^a^				
High	13 (16)	99 (63)	0.11 (0.05-0.24)∗∗∗	0.21 (0.07-0.65)∗∗
Moderate/low	66 (84)	59 (37)	Ref	Ref

COR: crude odds ratio for the unadjusted conditional logistic regression model; AOR: adjusted odds ratio for the backward stepwise conditional logistic regression model. ∗ denotes *P* < 0.05, ∗∗ denotes *P* < 0.02, and ∗∗∗ denotes *P* < 0.001. Ref: references category. ^a^Number of superscripts refers to dichotomized variables. High, daily or 1-2 times/week or ≥ 3 times/week, and moderate/low, never or less frequently (<3/month). ^¥^Variables adjusted for refrigerator, consumption of alcohol, consumption of white meat, consumption of red meat, consumption of processed meat, consumption of dairy product, preferences of the amount of fats/oils, preferences of amount of salts, preferences of condiments, consumption of green vegetables, and consumption of fruits.

## Data Availability

The data used to support the findings of this study are available within the manuscript.
